# Strategies of data layout and cache writing for input-output optimization in high performance scientific computing: Applications to the forward electrocardiographic problem

**DOI:** 10.1371/journal.pone.0202410

**Published:** 2018-08-23

**Authors:** Louie Cardone-Noott, Blanca Rodriguez, Alfonso Bueno-Orovio

**Affiliations:** Department of Computer Science, University of Oxford, Oxford, United Kingdom; Universidade Federal de Sao Joao del-Rei, BRAZIL

## Abstract

Input-output (I/O) optimization at the low-level design of data layout on disk drastically impacts the efficiency of high performance computing (HPC) applications. However, such a low-level optimization is in general challenging, especially when using popular scientific file formats designed with an emphasis on portability and flexibility. To reconcile these two aspects, we present a novel low-level data layout for HPC applications, fully independent of the number of dimensions in the dataset. The new data layout improves reading and writing efficiency in large HPC applications using many processors, and in particular during parallel post-processing. Furthermore, its combination with a cached write mode, in order to aggregate multiple writes into larger ones, substantially decreased the writing times of the proposed strategy. When applied to our simulation framework for the forward calculation of the human electrocardiogram, the combined strategy resulted in drastic improvements in I/O performance, of up to 40% in writing and 93–98% in reading for post-processing tasks. Given the generality of the proposed strategies and scientific file formats used, our results may represent significant improvements in I/O performance of HPC applications across multiple disciplines, reducing execution and post-processing times and leading to a more efficient use of HPC resource envelopes.

## Introduction

The optimization of high performance computing (HPC) codes is an area of active research, underpinning a continuous and cost-effective development of both established and emergent industrial and scientific sectors. As a representative example, the progress we are experiencing in computational medicine based on HPC applications is allowing the translation of mathematical models of physiological systems such as the heart to biomedical research and clinical practice. Within the field of cardiac electrophysiology, these include investigations on multiscale mechanisms of disease and lethal arrhythmias [1, 2], drug action [3, 4], electrical therapy [5, 6], causes of inter-patient variability in response to treatment [7, 8], the role of myocardial structure in modulating heart function [9, 10], identification of novel biomarkers for clinical diagnosis [11, 12], and the stratification of patients at high risk of sudden cardiac death [13], among others.

In scientific computing, substantial efforts to improve HPC performance are frequently placed on the optimization of the numerical solution of the underlying physical models. As in other disciplines, in cardiac electrophysiology this involves the development of strongly scalable solvers [14, 15], improved problem-specific preconditioners [16, 17], temporal and/or spatial adaptivity [18, 19], higher-order numerical schemes [20, 21], or the use of reduced models to alleviate model complexity [22, 23]. Additional areas of active HPC performance improvements include multithreading, load-balance, compiler optimization, or optimization at the application and operating system levels. Critically, scientific applications in large HPC systems often read and write vast amounts of data. However, much less attention is given in general to the input-output (I/O) optimization of these codes, frequently assumed as an inevitable burden with little scope for improvement, leaving I/O as a challenging factor in the overall performance of HPC applications [24].

Ideally, the first stage of I/O optimization in HPC applications should take place at the low-level design of the output structure, based on the most frequent access patterns to data. This is, however, a laborious task, in particular when using popular scientific file formats, designed with a focus on portability and flexibility (such as the HDF5 file format considered here [25]). To circumvent this complexity, the use of higher level analysis tools is usually preferred for HPC I/O optimization [26–29], commonly based on the profiling of communication, latencies and computation overheads in parallel applications. Other diagnostic tools also provide comprehensive summaries of data access patterns [30–32], which can then be used in later stages of I/O optimization. Additional middleware file formats with improved write and read performance have also been developed [33], although their acceptance in scientific applications still remains low.

To simplify such a delicate crafting process of low-level I/O optimization, in this work we present a general algorithm for the automatic design of data layout, solely based on the size of the dataset and one additional parameter, the target chunk size in bytes. When combined with a cached write mode, the new algorithm (applied to our simulation framework for the forward calculation of the human electrocardiogram) resulted in overall improvements in I/O performance of up to 40% in writing to disk, and between 93% to 98% in reading for different post-processing tasks. Given the generality of the proposed strategies and the scientific file formats used (HDF5 as a standard for portability and flexibility, and of widespread use among the scientific computing community), our methodology may be broadly applied to other scientific areas, yielding significant improvements in I/O performance of HPC applications and a more efficient use of HPC resources across multiple scientific disciplines.

## Materials and methods

### Bidomain equations in a bath

The bidomain equations [17] describe the evolution of the electrical activity in the heart (Ω_*h*_), surrounded by a conductive passive medium (i.e. the bath, Ω_*b*_). Two overlapping domains are assumed in the heart: the intracellular and extracellular domains, with respective potentials *ϕ*_*i*_ and *ϕ*_*e*_, whose difference provide the transmembrane potential (*V*_*m*_ = *ϕ*_*i*_ − *ϕ*_*e*_). The formulation of the problem is then given by the system of partial differential equations (PDEs):

$$\begin{array}{ccc} \chi (C_{m}\partial_{t}V_{m}+I_{ion})-\nabla \cdot (\sigma_{i}\nabla \phi_{i}) & =-I_{i}, & \text{in}\Omega_{h} \end{array}$$ (1)

$$\begin{array}{ccc} \nabla \cdot (\sigma_{i}\nabla \phi_{i}+\sigma_{e}\nabla \phi_{e}) & =0, & \text{in}\Omega_{h} \end{array}$$ (2)

$$\begin{array}{ccc} \nabla \cdot (\sigma_{b}\nabla \phi_{e}) & =0, & \text{in}\Omega_{b} \end{array}$$ (3)

$$\begin{array}{ccc} \partial_{t}u & =f(u,V_{m}), & \text{in}\Omega_{h} \end{array}$$ (4)

together with boundary conditions **n** ⋅ (*σ*_*i*_∇*ϕ*_*i*_) = 0 and **n** ⋅ (*σ*_*b*_∇*ϕ*_*e*_) = *I*_*e*_ on the heart and the external bath boundaries, respectively, where **n** represents the outward-facing unit normal. In the equations above, *σ*_*i*_ and *σ*_*e*_ are the intracellular and extracellular conductivity tensors, *σ*_*b*_ is the bath conductivity, *χ* is the surface-area-to-volume ratio, and *C*_*m*_ the membrane capacitance per unit area. The vector **u** contains cell-level variables (such as ionic concentrations and membrane gating variables), and *I*_*ion*_(**u**, *V*_*m*_) is the ionic current per unit surface area, as given by the cellular electrophysiological model **f**. The source term *I*_*i*_ is the intracellular stimulus per unit volume, whereas *I*_*e*_ is a stimulus current per unit area at the external boundary of the bath (zero in the absence of an external electrical field).

### Simulation environment

For the numerical solution of the bidomain equations we used Chaste (Cancer, Heart, and Soft Tissue Environment) [34, 35], an open-source electrophysiology solver package using the finite element method. Main dataset I/O used HDF5 version 1.8.14 on a Lustre filesystem. MPI was provided by the Cray MPT, based on MPICH 3. Simulations were performed on the ARCHER UK National Supercomputing service (http://www.archer.co.uk/). Our new HDF5 chunking algorithm with caching as described in this work is publicly available as part of the /io/src/ subfolder of Chaste’s open-source distribution (https://github.com/Chaste/).

### Benchmark problem

**Fig 1:**
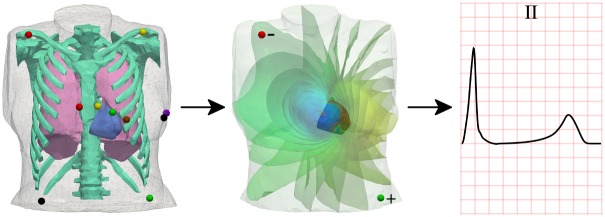
Illustration of the HPC scientific computing framework for which the I/O strategy was optimized in this work. From left to right: anatomically realistic human heart-torso mesh, also containing lungs and thoracic cage (left). The colored spheres indicate the location of the virtual electrodes for the calculation of the electrocardiogram, using standard (European) color-coding. Two double-precision floating-point numbers representing electric scalar fields in time and space are recorded at each node in the tetrahedral mesh (middle). These electric fields are finally post-processed to generate multiple time series (e.g. lead II as shown in the right panel), representing a simulated 12-lead electrocardiogram.

The HPC scientific computing framework for which the I/O strategy is optimized in this work is illustrated in Fig 1. The bidomain with bath equations were solved in an anatomically realistic human ventricular and torso (i.e. the bath, also containing lungs and bones) mesh. The combined heart-torso mesh has a total of about 3.25 million nodes and 19.4 million tetrahedra. A detailed description of model parameterization is provided in [36]. The ten Tusscher-Panfilov model [37] was used to describe human ventricular electrophysiology at the cellular level. For this mesh resolution with two double-precision outputs per printing time step (*V*_*m*_ and *ϕ*_*e*_), each printing time step requires storage of 8B × 3253316 × 2 ≈ 52.1MB.

The PDE time step was 25 *μ*s, with temporal adaptivity between consecutive PDE time steps for the numerical solution of the cellular electrophysiology model. For feasibility in generating the benchmark solutions, the printing time step was set to the PDE time step, and the total run time was 150 printing time steps. To include other types of data access, the post-processing of ventricular maps of activation times and peak transmembrane voltage were enabled, which involve reading from the main results dataset. Finally, all datasets were converted to VTK visualization files as an additional post-processing step.

## Results

### General considerations on HDF5 default data layout

At the hardware level, digital data storage is one-dimensional, so obviously there must be a mapping between the multidimensional datasets represented in HDF5 files and the disk. The default method simply serializes the in “row-major order”, which might or might not be suitable depending on the access pattern (the order in which each process accesses values in the dataset).

**Fig 2:**
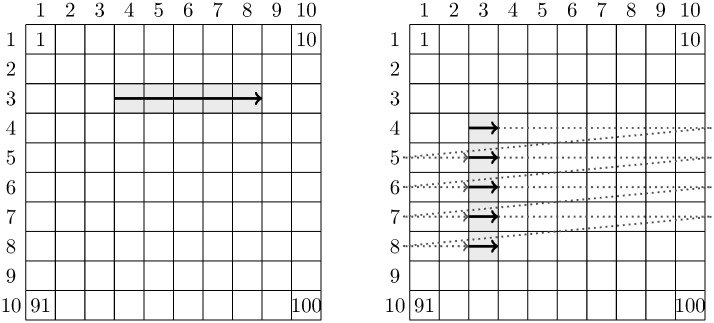
Default data layout in HDF5, and differences between reading a row and a column. A row (left) may be read efficiently because the elements are contiguous (solid arrow); reading a column (right) by contrast requires a disk seek (dotted arrows) before every read. The columns and rows of the dataset are labelled 1–10. Four data elements are labeled with their locations on disk (1,10, 91 and 100). Adapted from the HDF5 support webpage: https://support.hdfgroup.org/HDF5/doc/Advanced/Chunking/.

Suppose we have a two-dimensional 10 × 10 dataset using this default layout (Fig 2). In row-major order the data are serialized by rows, e.g. elements 1 to 10 (labelled) are contiguous on disk, and the same for the following rows. Using conventional matrix notation indexed from 1, if a process wants to read entries (3,4) to (3,8) inclusive, i.e. the 24^th^ to 27^th^ elements (shaded, top panel), then it can do this very efficiently by reading a contiguous region on disk (solid arrows). On the contrary, if a process wants to read entries (4,3) to (8,3) inclusive, i.e. the 33^rd^, 43^rd^, 53^rd^, 63^rd^, and 73^rd^ elements (shaded, bottom panel), then it must perform a number of relatively expensive disk seeks between each row (dotted arrows). Clearly, for good performance the data layout must be based on the access pattern.

Since data layout on disk has a significant influence on performance, HDF5 allows the specification of customized *chunk* shapes based on typical access patterns. A chunked dataset is divided into repeating units of the chunk dimensions, and space for each chunk is allocated contiguously on the disk. In the second example on column-reading above, we might utilize chunking by setting the chunk dimensions to the shape of a column in the dataset. With chunks that coincide with columns, the library would lay the data out on the disk column by column, so that any access from a column becomes a single, fast, contiguous read.

For cardiac applications, HDF5 datasets in our simulation package are three-dimensional objects, over time in the first dimension, nodes in the second, and variables in the third. A typical chunk has size {*C*_*t*_, *D*_*N*_, *D*_*v*_}, where: *C*_*t*_ depends on the number of printing time steps, *D*_*t*_; *D*_*N*_ is the total number of nodes; and *D*_*v*_ is the total number of variables. In other words, each chunk spans the dataset in the ‘nodes’ and ‘variables’ dimensions, with ceil(*D*_*t*_/*C*_*t*_) chunks in the time dimension. This strategy is fairly efficient for simulations with a small number of processors and nodes as each chunk will be relatively small and easy to cache. It is, however, poorly suited to large parallel applications as discussed below.

**Fig 3:**
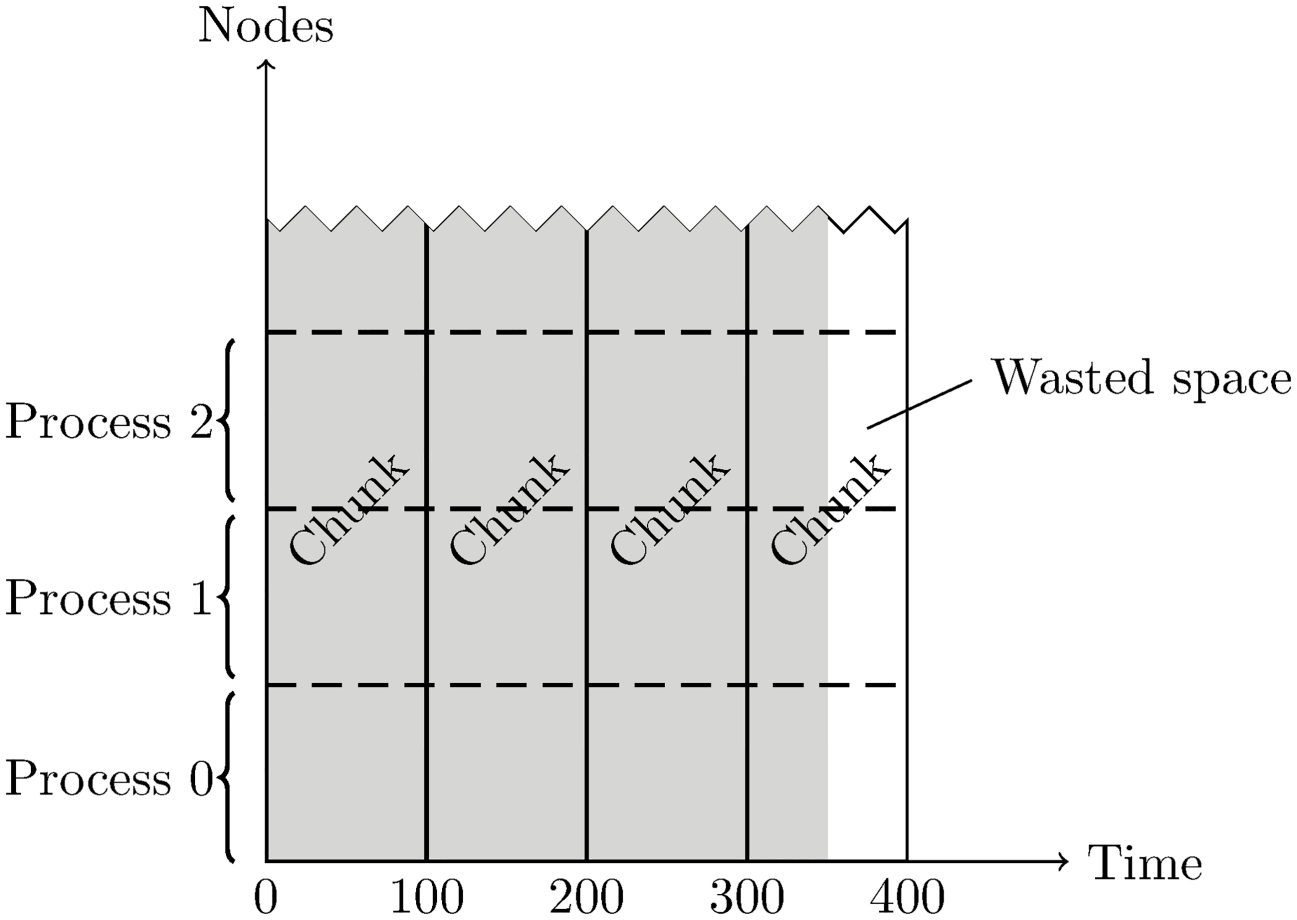
Typical chunk layout. This example depicts a dataset of size 350 in the time dimension and a large number in the nodes direction (grey). The chunks are of size 100 × *D*_*N*_ (solid lines), so 50 elements (12.5% of the file) are wasted at the edge (white). Each process is concerned with a slice of the dataset shaped orthogonally to the chunks (dashed lines). The third dimension has been suppressed for clarity, since the chunks and process boundaries span it.

Considering parallel performance, using chunks that span the node dimension is suboptimal due to the way the problem is partitioned across parallel processes. At the start of a simulation, the mesh is partitioned and each process is assigned a subset of the nodes that remains unchanged for the duration of the simulation. For simplicity, the nodes are reordered so that the nodes owned by each process are indexed contiguously. Using the earlier notation, each process will access a contiguous block of approximately size {*D*_*t*_, *D*_*N*_/*j*, *D*_*v*_}, where *j* is the number of processes (assuming an equal partition of nodes between processes). Recalling the chunk shape, we note that it is “orthogonal” to the regions owned by each process (Fig 3).

At this point it is necessary to briefly introduce two of the HDF5 drivers of our simulation package, and how they differ in reading and writing a chunked dataset. When writing, it uses the MPI-IO driver, which is specialized for parallel applications. The chunk shape has relatively little impact on writing because the MPI-IO driver uses direct access to the disk, and collective writes are used (designed to improve performance when writing from many processes to a single file by first concentrating data onto intermediate *aggregators*). When reading, however, (e.g. post-processing or generating visualization files) the default driver is used which uses standard POSIX operations. Unlike the MPI-IO driver, the default driver attempts to cache an entire chunk when data in it are accessed. Furthermore, the caching implementation in the default driver dictates that access can only be done on whole chunks. In other words, even if a process attempts to read just one entry from a chunk and it is possible for the chunk to be cached, then the process will read the entire chunk into the cache before continuing. If the chunk is too large to be cached then the cache is bypassed completely and direct access is used.

From the preceding paragraphs it becomes clear why using chunks that span the node dimension is suboptimal. First, consider an HDF5 reader being used to perform post-processing on a dataset in parallel. Each process is expected to access all the variables for all its nodes for all times. Depending on the size of each chunk compared to the size of the chunk cache there are two possibilities:

- If chunks are small relative to cache size, then when a process requests a value in its block it must first read the entire chunk into its cache, despite most of the nodes belonging to other processors and being therefore of no interest. This might yield acceptable performance if enough chunks can be cached and the access pattern is conducive, which is not the case in frequent post-processing tasks. For example, imagine the reader accesses all the time points for one node, then all the time points for the next node, etc. Unless all the chunks can be cached at once, the reader will be forced to read the entire dataset on every iteration, just to get the values for each node.
- If chunks are larger than cache size, then each process will access the dataset independently and directly. In this case, the potential performance improvement from using the cache is lost, but the requirement to read in whole chunks is dropped, possibly resulting in less disk activity. Still, for optimal performance the use of caching should be favored.

As mentioned above, writing to the HDF5 file is expected to be less affected by chunk settings than reading, since the MPI-IO driver only uses direct access with collective writes. Nevertheless, the chunk shape results in every chunk being accessed simultaneously by all processes (Fig 3), possibly resulting in increased library overhead to track modifications and maintain consistency between processes. A chunk layout more closely resembling the process boundaries would alleviate this issue.

### I/O optimization in large HPC systems

#### A new chunking algorithm

The most methodical way for optimal chunk design would be to set the chunk shape based on an analysis of the most frequent access patterns, within some chunk capacity limits. The chunk size is important because (1) disks are generally better at reading fewer, larger regions than more, smaller regions, and (2) it influences the size of chunk cache needed for good read performance. Unfortunately, two opposing modes of access coexist in our case at different stages of the simulation. When solving, the fastest varying dimension is the variable dimension, followed by nodes, and finally time. When performing post-processing, it is not uncommon to instead access the variables for each node over all time. An access-based approach might also result in highly problem- and/or machine-specific algorithms, that might show good performance in some applications at the expense of others.

Instead, a general algorithm was developed to set the chunk size based only on the size of the dataset and one parameter, the target chunk size in bytes (*T*_*B*_). For maximum generality and in order to ensure its applicability to any number of dimensions, the new chunk algorithm is designed to treat all dimensions equally. Another design requirement is that the chunk shape should result in high storage efficiency. HDF5 allocates the minimum integer number of chunks required to contain the dataset (recall Fig 3). Most conceivable chunk shapes *a priori* might therefore result in unacceptable amounts of wasted space at the edges of the dataset, because the chunk size is unlikely to be (close to) a factor of the dataset size in every dimension.

Central to the proposed solution is the variable “target size”, *T* (not to be confused with *T*_*B*_). First, for each dimension, the rounded-up division of the dataset size by the target size gives the minimum number of target-sized chunks that would span the dataset. Second, the rounded-up division of the dataset size by this number of chunks gives the actual size of chunk that is closest to the target size while still being close to a multiple. The problem then reduces to finding the target size that best satisfies the chunk size requirement in bytes. The solution can be written concisely as follows. Let the chunk and dataset sizes be vectors denoted by $\vec{C}$ and $\vec{D}$, respectively:

$$\vec{C}=(C_{1},C_{2},\ldots ,C_{N})$$ (5)

$$\vec{D}=(D_{1},D_{2},\ldots ,D_{N})$$ (6)

where *N* is the number of dimensions in the dataset (usually 3 for time, nodes and variables). We therefore get $\vec{C}$ by finding the largest *T* such that

$$8\prod_{i=1}^{N}C_{i}\leq T_{B}$$ (7)

$$\begin{array}{cc} C_{i}\leq D_{i}\; & \left(1\leq i\leq N\right) \end{array}$$ (8)

$$\begin{array}{cc} C_{i}=\left\lceil \frac{D_{i}}{\left\lceil D_{i}/T\right\rceil }\right\rceil & \left(1\leq i\leq N\right) \end{array}$$ (9)

where in Eq (7) represents the chunk size constraint (each element is 8 B), in Eq (8) limits the chunk to the dataset size in each dimension, and Eq (9) defines the chunk size given the dataset size and target size in such a way as to minimize wasted space.

**Algorithm 1** New HDF5 chunk size algorithm

1: $\vec{D}$              ▷ Dataset size in elements

2: $\vec{C}$              ▷ Chunk size in elements

3: *C*_*B*_ ← 0                ▷ Chunk size in B

4: *T* ← 0            ▷ Target chunk size in elements

5: *T*_*B*_ ← 128 × 2^10^          ▷ Target chunk size in B

6: ϒ ← False          ▷ Whether chunk spans dataset

7:

8: **function** SetChunkSize

9:  **while** (*C*_*B*_ < *T*_*B*_) &!ϒ **do** ▷While chunk is smaller than target

10:   Increment *T*

11:   $(\vec{C},C_{B},ϒ)\leftarrow$ CalculateChunkDims (*T*)

12:  **end while**

13:  **if** *C*_*B*_ > *T*_*B*_ **then**      ▷ If chunk has exceeded target

14:   Decrement *T*

15:   $(\vec{C},C_{B},ϒ)\leftarrow$ CalculateChunkDims (*T*)

16:  **end if**

17: **end function**

18:

19: **function** CalculateChunkDims (*T*)

20:  *C*_*B*_ ← 8              ▷ 8 B per element

21:  ϒ ← True

22:  **for** *i* in dimensions **do**       ▷ For each dimension

23:   *x* ← ceil (*D*_*i*_/*T*)

24:   *C*_*i*_ ← ceil (*D*_*i*_/*x*)

25:   *C*_*B*_ ← *C*_*B*_ × *C*_*i*_

26:   ϒ ← ϒ & (*x* ↔ 1)

27:  **end for**

28:  **return** $\vec{C},C_{B},ϒ$

29: **end function**

The method for solving the above is described in Algorithm 1. The dataset size ($\vec{D}$) and target chunk size (*T*_*B*_) are assumed as predetermined. The first while loop (line 9) increases the target size (*T*) and calculates the resulting chunk dimensions ($\vec{C}$) until the target size in bytes (*T*_*B*_) is reached, or the chunk spans the entire dataset (ϒ is True). Note that a binary search for *T* between 1 and $\max\left(\vec{D}\right)$ (for example) would be faster, but as invoked only once per dataset (resulting in a negligible overhead in overall wall times) this does not represent a significant increase in performance, and we chose to present the algorithm here in incremental form in the interest of clarity. After leaving the while loop, if *T*_*B*_ has been exceeded (line 13), the algorithm brings the size back below the target. Once given a target size in elements, the CalculateChunkDims function (line 19) calculates chunk dimensions that aim for the target size in each dimension while minimizing wasted space at the dataset edge as outlined above. First, it calculates the minimum number of chunks of size *T* that would be required to span the dataset (*x*, line 23). Then, given *x* chunks, it calculates the minimum number of elements required in each chunk to span the dataset (line 24). This function also calculates the actual chunk size in bytes (*C*_*B*_) and determines ϒ. The chunk size in bytes is the product of 8 B and all the elements of $\vec{C}$ (line 24). Finally, if *x* = 1 on every iteration, then one chunk spans the entire dataset and ϒ is True (line 26).

The choice of *T*_*B*_ depends on the problem size and the computer. The default value was set to 128 kB as this resulted in consistent performance in the small profiling tests that are run regularly to monitor performance. For large problems it was increased to 1 MB, in agreement with the default stripe size in Archer as discussed next.

#### Data striping

At the filesystem level, *data striping* is a common technique in HPC systems (including the *Lustre* filesystem in Archer) to increase data throughput by splitting files into segments and dividing the segments amongst multiple physical storage targets (e.g. hard disk drives).

**Fig 4:**
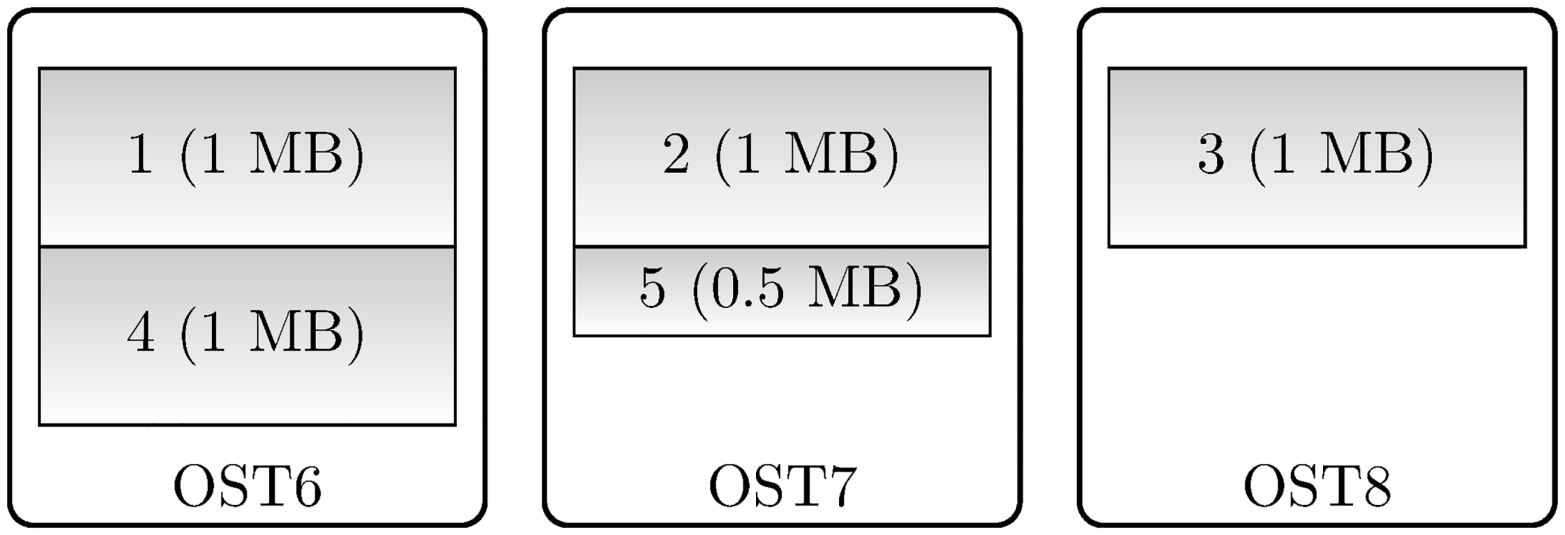
Data striping. This example depicts a 4.5 MB file striped across 3 OSTs with 1 MB stripe size. The first OST is chosen at random by the filesystem in order to load-balance, and in this example it is OST6. 1 MB stripes are then placed round-robin on each of the three OSTs, ending with a 0.5 MB stripe on OST7.

For performance improvements at this level, two parameters may be set on a file or directory basis: the stripe size (*S*) and stripe count (*c*). The former is the size (in bytes) of each stripe, whereas the latter is the number of Object Storage Targets (OSTs) over which to divide the stripes (see Fig 4). The system defaults on Archer are 1 MB and 4, respectively, and there are 48 OSTs at the time of writing this work, each capable of writing at roughly 500 MB/s.

Optimal values for *S* and *c* can only be found through experimentation. For reference, the Lustre documentation (see Section 18.2.1 in [38]) recommends a stripe size between 512 kB and 4 MB. Smaller sizes are not recommended ‘because the Lustre file system sends 1 MB chunks over the network’; more is not recommended because ‘stripe sizes larger than 4 MB may result in longer lock hold times and contention during shared file access’. Finally, the stripe size must be a multiple of the page size (enforced to a multiple of 64 kB for compatibility). The default stripe size on Archer (1 MB) is generally optimal and other values will not be considered here.

The stripe count *c* will be investigated below. For writes from many processors to a single file a large stripe count is recommended, but not too many counts as this results in extra overhead for diminishing returns. A starting point based on the Lustre documentation is to use approximately ‘1 stripe per GB’ to ‘1 stripe per 4 GB’ of file size. As an example, for 100 printing time steps of our benchmark problem (expected dataset size of ∼5.21 GB), *c* should probably be between 2 and 6. Another is to “load balance” by using an integer factor of the number of processors, such as one stripe per compute node so that each node gets one aggregator.

#### Cached writes

Regardless of the data layout used, the simulation results are written to the HDF5 file every print time step of simulation time. Recall from our benchmark description (see Methods) that one printing time step of data in our benchmark consumes about 50 MB on disk. Large HPC parallel file systems have good sustained throughput and are typically optimized for high bandwidth (such as the 500 MB/s per OST in Archer as mentioned above), but performance for small writes is much lower. They work best with a small number of large, contiguous I/O requests whereas small ones are generally discouraged. We should therefore expect several hundred 50 MB writes to show worse performance than, say, one 40 GB write.

The chunk cache provided by HDF5 might have been a viable answer, but it is currently not available when using the MPI-IO driver in write mode. The selected solution was to implement a memory cached mode in the HDF5 writer, whose constructor now takes an argument specifying if the cache will be enabled. This simplifies the implementation of our strategy over making the cache switchable, which would require extra logic like flushing the cache when switching. A new vector member with a reserved size of *C*_*t*_ × *N*_*n*_ × *N*_*v*_ acts as the cache, where *C*_*t*_ is the size of a chunk in the time dimension (as calculated by the new chunking algorithm), *N*_*n*_ is the number of nodes owned by a process, and *N*_*v*_ is the number of variables. Once the number of elapsed print time steps equals *C*_*t*_, each process writes the contents of its cache to the HDF5 file. As collective writes are still used, the library then takes care of consolidating the data onto aggregators as normal.

### Input-Output efficiency

#### Performance testing

In this section, the described data layout and cache writing strategies will be evaluated to investigate I/O efficiency. Their performance will be measured in the benchmark problem detailed in Methods, for both a small (8) and a medium (20) number of compute nodes (i.e. 192 and 480 cores, respectively) to test parallel scaling, and for three values of stripe count *c* (4, 24 and 42). Specifically, we compare:

1. Default chunks of size 41 in the time dimension, i.e. chunk size {41, 3253316, 2} (∼2 GB, the maximum single write size in ROMIO/MPI-IO).
2. New-style chunks of target size 1 MB, specifically {151, 434, 2} (1048544 B) as a result of applying Algorithm 1 to the dimensions of our dataset.
3. As in (2), but with caching enabled.

**Fig 5:**
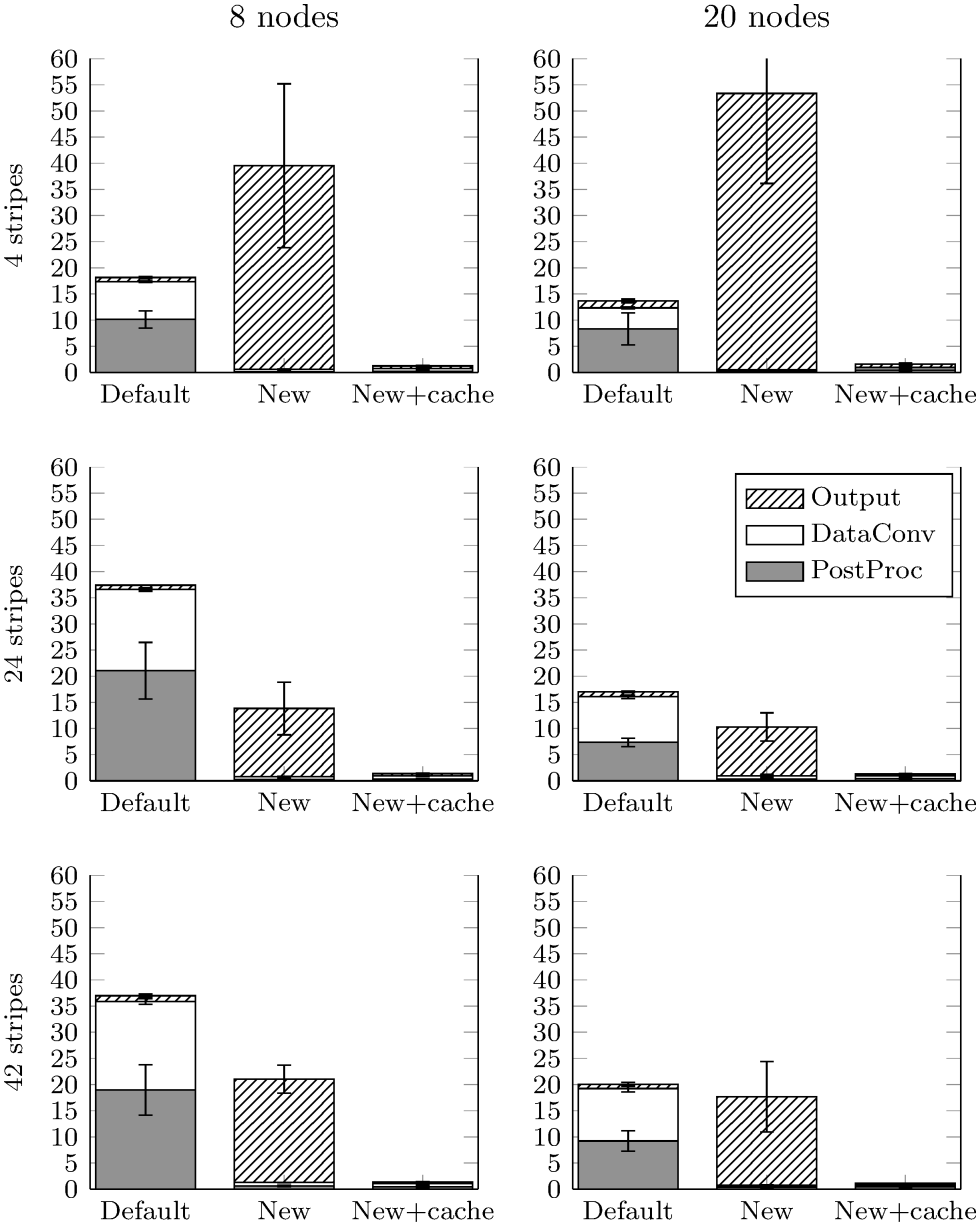
Summary of I/O results. Time spent writing to disk (Output), converting to VTK visualisation files (DataConv), and performing post-processing (PostProc) by each of the three methods: default-style chunks, new-style chunks, and new-style chunks with caching. The left and right columns represent results for simulations on 8 and 20 compute nodes, respectively. From top to bottom, the panels show results using stripe counts of 4, 24, and 42, respectively. Times are means from three repeats (in minutes), whereas error bars represent the S.E.M. Full data provided in S1 Table.

Benchmark results are shown in Fig 5. The three stacked bars in each panel correspond to the three strategies detailed above. The bars display the time spent (in minutes) in each of the following I/O categories: Output (writing to disk), PostProc (performing post-processing), and DataConv (HDF5 conversion to VTK visualization files). The three rows from top to bottom show results for each considered stripe count on the HDF5 file (4, 24, and 42, respectively). Finally, the left and right columns show results from 8 and 20 compute nodes (192 and 480 cores, respectively). Each simulation was performed three times in isolation to account for machine load. The times are presented as means and standard error of the mean (S.E.M.) of the three repeats. Performing additional repeats was unfeasible due to time and resource requirements.

The principal results of this study are as follows. In all cases, the default chunk layout (1) spent little time in Output and substantial time in DataConv and PostProc. The former point is likely due to the small number of chunks (just three), resulting in very little overhead when coordinating collective writes. The latter point, however, clearly illustrates the high cost of reading results from a poorly laid out dataset for additional post-processing tasks. Conversely, the new layout (2) spent the vast majority of time in Output and little in DataConv or PostProc. The writing of the resulting 7497 new style chunks is evidently slow, but the smaller, squarer chunks allow the post-processing and conversion steps to happen extremely quickly by leveraging the built-in HDF5 chunk-caching functionality. Moreover, the effect of enabling the custom chunk-writing cache on the new style method is striking (3). In this case, the benefits of the new data layout to DataConv and PostProc are retained, whereas the time spent in Output is reduced to under 30 s in all cases. Clearly the new algorithm with cached writes offers superior performance on Archer compared to the considered alternatives.

Fig 5 further illustrates the results for each method with respect to stripe and node counts, of importance for performance optimization. First, comparison by rows (stripe count effects) for the default chunk (1) illustrates increased DataConv times with 24 or 42 stripes compared to 4 stripes, both with either 8 or 20 nodes. Clearly, the DataConv process is unable to leverage the extra bandwidth offered by the large stripe counts, probably due to either an overhead of communicating with many OSTs, or technical advantage from concentrating on a small number of OSTs (e.g. internal OST caching). PostProc showed the same trend. In contrast, the uncached new chunks (2) were faster with 24 or 42 stripes than 4, showing performance benefits in parallel I/O. The severe bottleneck in Output is alleviated with a larger number of stripes, suggesting that the performance with 4 stripes is either limited by the OSTs or due to overwhelming the 4 threads assigned to aggregators. If we interpret the large error bars on Output as a sign of sensitivity to the machine load then the answer is probably the former. Finally, this method performed better with 24 stripes than 4 or 42, supporting the rule of thumb that a single large file written to by many processors should be striped, but not excessively, to avoid incurring large overheads. Another well-suited characteristic of the new-chunk cached method (3) for large HPC systems is its insensitivity to stripe counts, which alleviates optimization needs at the file system level, in particular for novice users.

Second, by comparing columns (node count effects), performance is improved for the default chunks (1) going from 8 to 20 nodes, both in DataConv and PostProc. This suggests that in spite of the sub-optimal I/O performance of these chunk layouts, the post-processing stage is somewhat able to utilize additional cores. The un-cached new algorithm (2), however, scales poorly at best, showing no significant difference between 8 and 20 nodes. In the case of 4 stripes, Output is still slow, perhaps exacerbated by the larger number of nodes. Yet again, there were no significant differences in performance for the cached new method (3) with node counts, highlighting its robustness for scalable applications.

The previous results also indicate that a strip count *c* of 4 simultaneously yields the best studied I/O performance for both the default chunks and new cached algorithm. This agrees with the initial estimate on that *c* should be between 2 and 6. A comparative summary of benchmark times for these 4 stripes on 8 compute nodes is presented in Table 1 for all the considered I/O strategies (see S1 Table for rest of stripes and compute nodes). These represent relative improvements in I/O performance of the new strategy over the default layout method of 40% in HDF5 writing to disk, 93% in post-processing, and 98% in HDF5 conversion to visualization files.

**Table 1:** Benchmark times for 4 stripes on 8 compute nodes. Results correspond to those illustrated in the top-left panel of Fig 5. Times are given in seconds (mean ± S.E.M.; full data provided in S1 Table.). The default chunk layout is fast in Output but slow in the other two categories. The new chunk shape is the opposite. With caching of writes enabled on the new shape the time spent in all three areas is low.

Data Layout	Output (s)	DataConv (s)	PostProc (s)
1. Default	49.5 ± 13.0	431.2 ± 8.0	608.7 ± 98.5
2. New	2333.5 ± 940.4	27.5 ± 3.2	10.0 ± 2.9
3. New + cache	29.9 ± 6.5	30.6 ± 5.0	14.6 ± 7.3

#### Alignment of new chunks with caching

As described in the presentation of our new chunking algorithm, any chunking algorithm is unlikely to produce chunks of exactly the target size. In addition, chunks are located by default at irregular locations within the file. Together, these two statements imply that a given chunk is unlikely to align perfectly with the stripe boundaries of the file. In such circumstances, accessing a chunk requires reading stripes from more than one OST. For example, if the stripe size and chunk target size are 1 MB and the true chunk size is slightly less than 1 MB, then reading a chunk is likely to involve requests to two OSTs. When the chunk and stripe size are similar, it might therefore be preferable for each chunk to be padded slightly with empty space so that each chunk starts on a stripe boundary. Note however that such an approach should be used with caution in order to avoid excessive wasted space.

The benchmark problem was used to test the performance with and without alignment (as implemented natively in the HDF5 library) in the new cached chunking algorithm. Results (mean ± S.E.M., in seconds) are shown in Table 2 for 110 and 113 repetitions of the unaligned and aligned cases, respectively.

**Table 2:** Benchmark times for the new chunking algorithm. Cached writings are used (4 stripes on 8 compute nodes), with and without HDF5 alignment set to the stripe size. Times given in seconds (mean ± S.E.M.). Full data provided in S2 Table.

Method	Output (s)	DataConv (s)	PostProc (s)
Unaligned	20.7 ± 0.4	30.2 ± 1.3	7.5 ± 0.2
Aligned	23.8 ± 1.2	31.0 ± 1.3	8.4 ± 0.2

Whereas HDF5 alignment did not substantially affect the overall performance of the new method, no improvements were attained in any of the considered I/O categories. A possible explanation for this is that the processes regularly read and write across chunk boundaries (i.e. the partition boundaries rarely fall exactly on chunk boundaries), so placing each chunk into its own stripe rarely results in a reduction in the number of OSTs accessed. Enabling alignment might also introduce small gaps into otherwise contiguous data, reducing performance slightly. The theoretical advantage of aligning chunks to stripes might however become apparent in larger problems.

## Conclusion

In this work, we have presented significant improvements in the I/O performance of our electrocardiogram-simulation framework in large HPC infrastructures, particularly in the challenging and frequently neglected areas of data writing, post-processing, and data conversion. A general algorithm for the efficient design of data layouts in HDF5 files (as a leading scientific file format for data storage and portability) was developed, and further optimized using cached writes. The efficiency of the resulting I/O strategy with respect to native concurrent layouts has been shown, independently to stripe and node count effects in large HPC filesystems, as well as to data alignment within the resulting files. Furthermore, as a single parameter (the target chunk size in bytes) is responsible in our algorithm for the low-level design of the underlying datasets regardless their number of dimensions, this guarantees a maximum generality and its applicability to other scientific areas beyond the one considered in this work.

The most substantial contribution is the method in which HDF5 files are written to disk, including the design of a novel low-level data layout independent to the number of dimensions in the dataset. Two actions are central to this I/O strategy. Firstly, the data layout (the so-called chunk shape) was modified to improve efficiency when reading small amounts of data, which is common in large HPC applications using many processors. This yielded a significant reduction in times for post-processing of simulation results and their conversion to other visualization formats, which are common scientific requirements across disciplines. A side effect of the new data layout was an increase in the output times required for the writing of results to the HDF5 file, due to the nature of storage systems in large HPC systems. This was overcome by implementing a cached write mode which bundles multiple small writes into larger ones, substantially reducing the aggregate writing times. The overall result was a drastic reduction in the time spent in all I/O stages of our simulation framework, with relative improvements over default HDF5 layouts of 40% in writing, 93% in post-processing, and 98% in data conversion.

Previous efforts have also been deployed for the optimization of I/O performance in HPC applications. Of particular relevance for our work are write-optimized middleware systems, such as ADIOS (Adaptable IO System, [39]) or PLFS (Parallel Log-structured Filesystem, [40]). These high-level I/O Application Programming Interfaces (APIs) allow for a more aggressive writing and efficient reordering of data locations in the case of ADIOS, and for a decoupling of concurrent writes to improve the speed of checkpoints in the case of PFLS, resulting in up to 100 × improvements in writing in selected applications [40, 41]. Importantly, these write-optimized APIs have been also shown to not penalize read speeds [33]. However, they both introduce intermediate file formats that require conversion for analysis to standard scientific formats, or to be mounted as stackable filesystems on top of an existing parallel filesystem.

On the contrary, the simplicity of the I/O strategies presented in this work, solely based on the size of the dataset (independent to its number of dimensions) and the straightforward implementation of a cached write mode, could easily be incorporated into codes using popular scientific file formats like HDF5, which has a history of optimization on popular HPC platforms [42]. This would alleviate the need of using intermediate API layers and the associated additional complexity to end users, while resulting in important savings in writing, reading and post-processing times in scientific applications.

For applications involving mesh adaptivity, an inherent limitation of the HDF5 file format is that the chunk size is set at the dataset creation time and cannot be changed later, which forces the use of a fixed chunk size. Based on our results for fixed chunk sizes, we hence still expect an increased I/O efficiency for the new designed chunks compared to the default HDF5 layout in the presence of adaptivity. Such investigations (including the estimation of an optimal chunk size) fall however beyond the scope of our present work. In addition, our benchmark experiments were performed using a single (Lustre) parallel I/O environment. Although our cached results demonstrate almost complete independence to the number of stripe counts (see Fig 5), which in turn minimizes sensitivity to the choice of this parameter as a common technique to increase data throughput across multiple HPC filesystems, the evaluation of our methodology in other parallel environments also constitutes an interesting aspect for future research.

In conclusion, given the generality of our I/O strategies and file formats used, the improvements presented in this work might enable a more efficient use of HPC resources and accelerated progress in multiple areas of scientific research. This may allow researchers to achieve a wider range of functionalities using standard scientific file formats, and therefore more complete simulation frameworks, within tolerable HPC resource envelopes.

## Supporting information

S1 Table Benchmark results for considered data layouts. I/O times for data writing to disk, data conversion, and post-processing in default-style chunks, new-style chunks, and new-style chunks with caching.
(XLSX)

S2 Table Benchmark results under HDF5 alignment. I/O times for data writing to disk, data conversion, and post-processing in new-style chunks with caching, with and without HDF5 alignment.
(XLSX)
